# Body Size Misperception and Overweight or Obesity among Saudi College-Aged Females

**DOI:** 10.1155/2018/5246915

**Published:** 2018-05-21

**Authors:** Jumanah Albeeybe, Abdulaziz Alomer, Tasneem Alahmari, Nawal Asiri, Reema Alajaji, Reem Almassoud, Hazzaa M. Al-Hazzaa

**Affiliations:** ^1^College of Medicine, King Saud University, P.O. Box 7805, Riyadh 11472, Saudi Arabia; ^2^Department of Orthopedics, King Saud University Medical City, King Saud University, P.O. Box 7805, Riyadh 11472, Saudi Arabia; ^3^Lifestyle and Health Research, Health Science Research Center, Princess Nourah Bint Abdulrahman University, P.O. Box 93216, Riyadh 11673, Saudi Arabia

## Abstract

The aim of the study was to investigate the associations between perceived and desired body size and overweight and obesity among college-aged females. A multistage stratified cluster random sample was used to select 907 healthy females from a major Saudi public university. The Stunkard Figure Rating Scale (FRS) was used for body size assessment. Overweight/obesity classification was based on BMI less than or equal to/greater than 25 kg/m^2^. Overweight plus obesity prevalence was 28.1%. There were significant differences between females with overweight/obesity and those without overweight/obesity in both perceived and desired body size scores. Compared with only 4% of females without overweight/obesity, 37% of the participants with overweight/obesity scored higher than five (median) in the FRS. The perceived body size correlated more strongly with many of the selected variables than did the desired body size, especially with BMI (*r*=0.679; *p* < 0.001), body weight (*r*=0.652; *p* < 0.001), and weight loss attempts (*r*=0.466; *p* < 0.001). Also, there was a significant relationship between BMI and weight loss attempts (*r*=0.370; *p* < 0.001). BMI and weight loss attempts appear to predict the perceived body size and the discrepancy between perceived and desired body size scores. Psychosocial and lifestyle factors that might influence female's body misperception need to be addressed in future studies.

## 1. Introduction

The prevalence of obesity is increasing worldwide [1]. Such an increase in the prevalence of obesity represents a major public health challenge [1, 2]. Obesity is well recognized to associate with variety of chronic diseases [3]. Women's obesity in Saudi Arabia is considered among the highest in the world [4]. Efforts to combat this problem need to consider all behavioral aspects that are associated with overweight and obesity. One important component of behavioral change is the recognition of excess body weight and its related health risks. Perceiving their own body size correctly is considered an important motivation for taking action and engaging in healthy lifestyle behaviors [5]. Body size perception refers to the image that people form of their physical appearance in their mind and represents the degree to which individuals are satisfied with their body weight and shape. It is a multidimensional concept involving and influenced by biological, psychological, social, and cultural determinants [6].

Body dissatisfactions are common among college-aged women, as many female students tend to perceive their bodies to be considerably heavier than their actual body weight status [7, 8]. In a large sample of participating American females between 11 and 21 years, it was found that 25.1% of normal-weight females perceived themselves as overweight [9]. In addition, a 13-year cohort follow-up study revealed that women with obesity who perceived themselves as normal weight gained a considerable amount of weight, while those who correctly perceived themselves as obese had lost a significant weight from their excess body weight [10]. On the contrary, overweight perception was found to associate with higher risk of future weight gain among adults from the United States and UK [11], while body size satisfaction appears to provide some protection against excessive weight gain and binge eating [12].

Variation in body dissatisfaction related to weight status by ethnic groups has also been recognized, as African American with overweight/obesity [13] and Arab [14] adolescents who perceived themselves as underweight were more likely to gain body weight. The high occurrence of negative BI among young females represents a real concern and can influence weight status and development of body shape dissatisfaction. BI dissatisfaction has been shown to associate with a number of unhealthy behaviors such as unsafe dieting, lower self-esteem, eating disorders, and depressive symptoms [15–17].

Cultural and social influences appear to impact body image perception, weight norms, obesity, and body shape dissatisfaction and may consequently increase susceptibility to eating disorders [18–20]. Traditional cultures are thought to have protective effects against the development of eating disorders, whereas exposure to western culture may increase such risks through exposure to values communicated by media and peer contacts [21, 22]. Indeed, culture-specific influences such as faith was recently shown to have a protective effect against body dissatisfaction and excessive dieting in Bulgarian women [23]. Further, sub-Saharan African women tend to accept their body size more than white females [22]. In some African countries, like Ghana, although overweight was considered undesirable by most adult women, some weight gain was admired, considered beautiful by women and was perceived as a sign of financial prosperity by their family and friends [24]. Among a nationally representative US sample, it was shown that body weight perception did vary by sex and race/ethnicity, and that American White girls were more likely to perceive objectively lower weights as “about the right weight” compared to young African Americans, Asian American, or Native Americans girls [25]. This may explain why normal-weight young white females in the USA, who were more likely to engage in dieting, use diet products and exercise to lose weight than normal-weight African American or Hispanic females [26].

There is amble evidence accumulated within the last decade indicating that body weight concerns, dissatisfaction, and/or eating disturbances are fairly widespread among young Arab women [27–29]. A recently published research involving a large sample of Arab females attending university in five Arab countries including Bahrain, Egypt, Jordan, Oman, and Syria revealed that one-third of the females were dissatisfied with their body weight status [30]. In addition, earlier reports on body size perceptions among young women from an urban health center in eastern Saudi Arabia showed that a substantial proportion of them either overestimated (28.6%) or underestimated (28.9%) their actual body weight [31]. However, due to recent globalization and adoption of western lifestyle and eating behaviors by many developing countries including Saudi Arabia and neighboring Gulf States, the attitudes towards body weight and weight perception may have changed, shifting to a preference for thin body size [14, 32, 33]. Understanding different factors that promote body satisfaction across racial and ethnic groups is an important tool that may facilitate adopting healthy lifestyle behaviors, improve effective obesity prevention interventions, and prevent body weight dissatisfaction and eating disorders. With this background, the aim of the present study was to investigate the associations between self-perceived and desired body size and levels of overweight or obesity among a representative sample of Saudi college-aged females. Information on body size perception in relation to overweight and obesity among young Saudi females has been fairly limited.

## 2. Methods

### 2.1. Participants and Study Design

Saudi college-aged females who were attending King Saud University (KSU) during the academic year 2015/2016 comprised the study population. KSU is a major comprehensive public university located in the city of Riyadh, the capital of the country. Students attending King Saud University come from all regions of the country. A multistage stratified cluster random sampling technique was used to select the desired sample. In the first stage, a systematic random sampling procedure was used to select three colleges from within three major types of colleges within the university; humanity, health sciences, and science and computer science colleges. Then, female students within each selected college were chosen randomly through cluster sampling as the class or course group represented the cluster. The selection process made certain to include classes from each of the 4 or 5 years of study in each college.

Assuming an obesity prevalence of 20% among college students, with a confidence level of 95% and an amount of error of 2.5%, the sample size was determined as 783 students. Adding to that 15% for clustered design effect and missing data, the target sample size was calculated as 901 participating females. The ethical committee in the College of Medicine at King Saud University approved the study protocol and procedures. Written consent forms were also obtained from all the participating females.

Body weight was measured to the nearest 100 g, with minimal clothing and without shoes, using a calibrated portable medical scale (Seca Scale Model 770, Seca, Hamburg, Germany). Height was measured to the nearest cm with the subject in the full standing position without shoes using a calibrated portable measuring rod. The body mass index (BMI) was calculated as body weight in kilograms divided by squared height in meters. Participants were classified as overweight/obese (BMI ≥ 25 kg/m^2^) or nonoverweight/nonobese (BMI < 25 kg/m^2^).

### 2.2. Body Size Assessment

For the body size assessment, we used the Stunkard Figure Rating Scale (FRS) images [34]. The body image scores were calculated in two ways: first, the student's perception of her actual body size based on the 9 silhouettes figures (called perceived body size), and second, the image that the student desires to look like from the same 9 figure images (called desired body size). Then, each figure was given a corresponding BMI score according to a previous study that has linked the actual BMI values to the FRS BI scores using a large sample size of adult females [35]. Finally, three main discrepancy (difference) scores were calculated: first, the discrepancy between perceived and desired body size scores, which represented body dissatisfaction; second, the discrepancy between the actual BMI and the BMI value corresponding to the perceived body size score, which represented body size misperception; and third, the discrepancy between the actual BMI and the BMI value corresponding to the desired body size score.

### 2.3. Data and Statistical Analysis

The data entered into a coded SPSS data entry sheet were checked and analyzed by IBM SPSS software, version 22. Descriptive statistics (mean, SD, or frequency) were obtained for selected anthropometric and demographic variables. Females with overweight/obesity versus those without overweight/obesity were classified based on BMI values of less than or equal to/greater than 25 kg/m^2^. In addition, cross tabulation with chi-square tests was used to calculate the proportions (percentage) of perceived and desired body size scores selected by Saudi females with or without overweight/obesity. The *T*-test with independent samples was used to test the differences between participants with overweight/obesity versus those without overweight/obesity in the following body size discrepancy scores: discrepancy between perceived and desired body size scores, discrepancy between BMI values corresponding to perceived and desired body size scores, discrepancy between actual BMI values and BMI corresponding to perceived body size scores, and discrepancy between actual BMI values and BMI corresponding to desired body size scores. The relationships between selected variables were also tested using Pearson's correlation coefficients. Finally, linear regression analysis with stepwise method was used to predict the perceived body size scores, desired body size scores, and the discrepancy between perceived and desired body size scores, as dependent variables with anthropometric and demographic variables as predictors. The following variables were entered in the regression analyses: age, weight, height, BMI, father education, mother education, family income, weight-loss attempt, number of obese kids in the family, and parents' obesity. In all of the tests performed, we used alpha level of <0.05 as the level of significance.

## 3. Results

The total number of the sample was 907 healthy females. The majority (59.6%) of the college-aged females are of normal weight; however, overweight (20.4%) plus obesity (7.7%) prevalence reached 28.1%, while the proportion of females with underweight was 12.3%.  Table 1 presents the participants' characteristics as a whole and relative to overweight/obesity status. The females with overweight/obesity are slightly older than those without overweight/obesity. There were no significant differences between the two groups in body height, father education, mother education, or family income. However, females with overweight/obesity have significantly more obese siblings in the family and more parental obesity. There were significantly more attempts to lose weight by the Saudi females with overweight/obesity compared with those without overweight/obesity.

The proportions (%) of perceived and desired body size scores selected by the Saudi females with or without overweight/obesity relative to the 9 FRS scores as well as the corresponding normative and actual BMI values are presented in Table 2. There were significant differences between participants with overweight/obesity and those without overweight/obesity in both perceived and desired body size scores; however, the perceived body size scores are much more pronounced in the females with overweight/obesity compared to those without overweight/obesity. 37% of the girls with overweight/obesity as opposed to only 4% of those without overweight/obesity scored higher than a score of 5 (median score) on FRS. In addition, females with overweight or obesity who gave themselves a score of 5 or higher on the Stunkard Figure Rating Scale more closely resembled their actual BMI values than those who perceived themselves as having less than a score of 5. The overwhelming majority (>87%) of females (with and without overweight/obesity) desired a score of lower than 5, which corresponds to a BMI value less than 26.2 kg/m^2^ [35]. In general, higher proportion of females with overweight/obesity underestimated their body size scores compared to those without overweight/obesity.

Table 3 shows perceived and desired body size scores, actual BMI, and some computed discrepancy scores among Saudi females with overweight/obesity and those without overweight/obesity. There are significant differences between the two groups in each of these measured and calculated variables. Of particular interest is the pronounced differences between the actual BMI and BMI value corresponding to the desired body size in the females with overweight/obesity (−8.5 ± 5.2 or −28.9%) compared to those without overweight/obesity (−0.30 ± 3.1 or −1.4%). When calculating the BMI values corresponding to perceived body size scores based on the normative BMI from a previous study [35], females with overweight/obesity underestimated their actual BMI by –2.5 ± 2.5 kg/m^2^ (8.5%), while those participants without overweight/obesity slightly overestimated their BMI by +0.40 ± 2.5 kg/m^2^ (1.9%).

The correlation coefficient analyses of selected variables showed that perceived body size scores correlated significantly with the BMI (*r*=0.679; *p* < 0.001), the BMI value corresponding to perceived body size (*r*=0.968; *p* < 0.001), and the number of weight-loss attempts by the participating females (*r*=0.466; *p* < 0.001), whereas desired body size scores revealed significant associations with BMI (*r*=0.089; *p*=0.008) and the BMI value corresponding to desired body size (*r*=0.951; *p* < 0.001). In addition, there is a significant moderate relationship between the BMI and the number of weight-loss attempts (*r*=0.370; *p* < 0.001). Family income did not show any significant relationship with the perceived BMI (*r*=0.039; *p*=238) or with desired body size (*r*=−0.051; *p*=0.123). In addition, perceived body size scores have low but significant correlation with parent obesity (*r*=−0.107; *p* < 0.001).

Finally, Table 4 presents the results of multiple linear regression analyses for the prediction of some selected dependent variables (perceived and desired body size scores and the discrepancy between perceived and desired body size scores). The BMI and the number of weight-loss attempts appear to predict the abovementioned dependent variables. Model 2 of these predictors shows that the common variances when predicting perceived body size and the discrepancy between perceived and desired body size scores, using BMI values and the number of weight loss attempts as predictors, amounted to 51% and nearly 47%, respectively.

## 4. Discussion

The present study reported perceived and desired body size findings relative to overweight and obesity among a large and representative sample of Saudi college females from a public multidisciplinary higher education institute in central Saudi Arabia, where students come from all over the country. The overweight plus obesity rate among the study's participants was 28.1%. Such an average value for our college female sample is much lower than the national prevalence of overweight plus obesity among Saudi adults, which was reported to be 61.5% [4]. However, our sample was much younger than that reported in the national prevalence. In addition, the findings of this study showed a certain extent of imprecise perception of body size in both groups of college females. A considerable proportion of the Saudi females with overweight/obesity underestimated their body size scores, while a smaller proportion of those without overweight/obesity underestimated their body size scores. Such observed findings seem consistent with many previous studies [14, 33, 36–39]. In addition, a study conducted on university students from 7 European countries showed that weight ideals were rather uniform across the European countries, with female students being more likely to perceive themselves as too fat at normal BMI values [40]. Interestingly, Korean women had lower discrepancies between their current and ideal (desired) body weight than US women had, leading to the conclusion that Korean women were more satisfied with their body weight [41].

Previous local studies about body image perception among young Saudi females had mixed results. A cross-sectional study involving 663 university female students from Southeastern Saudi Arabia showed a 23% agreement between the perceived and desired body image [33]. However, in our study of college females, the common variance between perceived and desired body size was only 8%. Another study in Eastern Saudi Arabia that used the Body Shape Questionnaire and included 368 female and male students from King Faisal University revealed that only 9.4% of females with overweight plus obesity were dissatisfied with their body shape. Interestingly, the study showed that more females in the normal weight were dissatisfied (22.7%) with their body shape [32]. Furthermore, the majority of the Saudi women attending gymnasium classes in the city of Hail in Northern Saudi Arabia identified their own body shape as being overweight, but they chose the “normal weight” figures as the image they wanted to look like [42]. In the neighboring state of Bahrain, a significant discrepancy between adolescents' perception of body weight and actual BMI was found. There was a propensity for Bahraini teenagers with overweight and obesity to underestimate their weight status, and more than half of the girls expressed dissatisfaction with their current body weight [14]. In a comparative study involving university students aged 17–32 years from five Arab countries (Bahrain, Egypt, Jordan, Oman, and Syria), it was found that 65–87% of Arab college females preferred a thinner body size or a figure rating of 1–3 in the FRS [29].

Results of the present study showed that there were significant differences between perceived and desired body size scores relative to female's overweight and obesity status. Of particular interest is the pronounced differences between the actual BMI and BMI value corresponding to desired body size among college females with overweight/obesity (8.5 ± 5.2 kg/m^2^) versus those without overweight/obesity (0.30 ± 3.1 kg/m^2^). Similar findings were reported for young females from diverse ethnicity in different countries around the world, showing significant differences between their actual and desired (ideal) body shape and resulting in a fairly high level of body size dissatisfaction [38, 39, 43–45]. Analysis of data from large national prospective cohort in the United States involving 16,882 participants aged 9–18-year-olds showed that girls above 50th BMI percentile reported greater dissatisfaction than girls below the 50th BMI percentile [37]. The study also found that younger youth with overweight and obesity were reporting the greatest body weight concern; however, among older females, healthy weight became increasingly associated with greater weight and body shape concern [37]. It is interesting that body size misperception may occasionally provide some protection against future weight gain, as overweight or obesity misperception among youth from the United States was found to predict lesser future weight gain [46]. In addition, young women with obesity who correctly perceived themselves as obese had lost significant weight from their excess body weight [10]. Moreover, overweight adolescent girls with body dissatisfaction gained more body weight five years later [47].

Having a positive body image is important for young females because it is believed to affect their self-esteem. Research has found a fairly strong relationship between low self-esteem and body-size dissatisfaction [48] Negative body image was also shown as a strong predictor of eating disorders [16]. Using the exercise and self-esteem model as a guiding framework, results support the notion that, to improve body image, perceived changes to the body size are more important than actual changes [49]. However, it has been argued that body weight perception is independent of the actual weight status, as the participants who have been given values of their body weight before rating their body shape still reported inaccurate perceptions of their body size [36]. Moreover, a qualitative study conducted on young women from two different weights revealed that women experience a significant emphasis on overweight issues with a trend towards appearance rather than health [50]. The young women showed strong views on the cultural definitions of normal body weight and described the conflict between cultural norms and their perceptions of body size and health as a likely factor negatively influencing well-being, weight-balance, and motivation to alter lifestyle [50]. Indeed, in the Internet era, females in particular are exposed to mixed messages about body image promotion. One message is coming from the global mass media and weight loss industries and another one from health awareness movements that is encouraging females to accept a healthy body image regardless of body shape [51]. In either direction, such a message may have a negative or positive influence on future control of body weight and obesity prevention among females.

The current findings revealed a positive relationship between the BMI values corresponding to perceived body size scores and the actual BMI values (*r*=0.697; *p* < 0.001). Such normative values linking the actual BMI to the FRS body image scores have been previously established for adult females [35]. Our results also indicated a significant moderate association between perceived body size rating and the number of weight-loss attempts. This may reflect the notion that some females who were trying to lose weight appeared more realistic about perceiving their body size, and this may play a role in weight control behaviors. Parental obesity also had a low but significant correlation with the perception of body size, which may point out to the influence of home environment on the perception of body weight images. In addition, the present findings did not show any significant relationship between family income and perceived or desired body size. However, results from the first international body project survey about body weight ideals and body dissatisfaction in 10 major world regions revealed that body dissatisfaction and desire for thinness are commonplace in high socioeconomic status settings across the world [52]. In contrast, a research studying African American adolescents concluded that socioeconomic status is correlated neither with the BMI value nor with the self-perception of body weight [53]. Furthermore, in a comparative study between three countries, the perceived body image and weight control behavior differed among adolescents in Lithuania, Croatia, and the USA, and the correlation between body size and BMI was 0.85 [54]. Similar correlation (*r*=0.85) was reported between body size, using Stunkard nine-level figures, and BMI in American young women between the ages of 18 and 44 years [55]. The discrepancy between ideal and actual body size was also found to be significant in Dutch young women with Morocco or Turkish ethnicity, and that the perceived body size was correlated with the BMI in these females, as Spearman's correlation coefficient was at 0.73 [39].

The results of multiple linear regression analyses indicated that the BMI and the number of weight-loss attempts, but not family income or parents' education levels, appear to strongly predict perceived body size scores as well as the discrepancy between perceived and desired body size scores. However, a local cross-sectional study involving university female students from Southeastern Saudi Arabia showed physical activity, family income, fathers' level of education, numbers of owned cars, and the kind of residence were positively associated with the desire to be thin, whereas the number of sisters and the number of household cars were correlated positively with the desire to be heavy [33]. Further, a study conducted on adult residents of Nairobi slums indicated that the major predictors of underestimation of body weight among overweight and obese respondents were age and sex, but marital status and education were not significantly associated with the likelihood of BMI underestimation [56]. Among female students attending Kuwait University, the perceived body image and a desire to be thinner were strongly related to body-image dissatisfaction, and the addition of age, marital status, physical activity, dieting behavior, parental education, and family size did not significantly alter the findings [57].

### 4.1. Strengths and Limitations of the Study

The present study has several strengths and limitations. The strengths of the current investigation include having a large and representative sample of Saudi college-aged females selected from a diversified and large public university and the use of well-validated and widely used instruments for the assessment of the body image (body size). A further strength of our study is that weight and height values were actually measured and not reported by the participants. On the other hand, the first limitation of the present study is the cross-sectional design, which cannot imply a casual relationship between body size rating and the BMI or which factor influenced the other first. Second, the selected body size measure in the present study is not a comprehensive one, and it provides a partial assessment of women's body size concerns. Third, our data were generated from a sample of college-aged females living in an urban area, and therefore the findings may not be generalized to other sittings or segments of the Saudi population, such as working women, less-educated females, different age groups, or those living in rural areas.

## 5. Conclusion

Our findings indicate that a considerable proportion of college females, especially those with overweight or obesity, have underestimated their perceived body size. This means that female college students with a BMI ≥ 25.0 kg/m^2^ seem more likely to present with body size dissatisfaction than those peers without overweight/obesity. Such findings may point out to the sociocultural influences and media pressure that emphasized body thinness among young females. Body size misperception among Saudi females with obesity is concerning. Therefore, it is recommended that psychosocial and lifestyle factors that might influence body misperception among Saudi females to be addressed in future studies. In addition, efforts to prevent body size dissatisfaction needs to begin early before the college age as part of both obesity and eating disorder prevention programs. Finally, our study contributes to the small existing literature on body image perception in relation to obesity in Saudi young females.

## Figures and Tables

**Table 1:** Participants' characteristics according to overweight/obesity levels.

Variable	All (*N*=905)	Overweight/obese (*N*=254)	Nonoverweight/nonobese (*N*=651)	*p* value^*∗*^
Age (years)	20.9 ± 1.8	21.4 ± 1.3	20.8 ± 1.5	**<0.001**
Weight (kg)	58.8 ± 14.0	74.5 ± 15.1	52.7 ± 7.2	**<0.001**
Height (cm)	158.8 ± 5.7	159.0 ± 7.8	158.7 ± 5.6	0.462
Body mass index (kg/m^2^)	23.3 ± 5.1	29.4 ± 5.1	20.9 ± 2.4	**<0.001**

Father's education (%)				
High school or less	—	44.9	41.5	0.755
University degree	—	38.6	39.5	
Postgraduate degree	—	16.5	19.0	

Mother's education (%)				
High school or less	—	48.1	53.6	0.349
University degree	—	43.2	40.2	
Postgraduate degree	—	8.7	6.2	

Family income (%)				
5000 SR or less	—	5.5	7.4	0.260
5001–10,000 SR	—	15.4	10.1	
10,001–15,000 SR	—	13.8	16.7	
15,001–20,000 SR	—	16.1	17.5	
20,001–25,000 SR	—	15.0	15.2	
25,001+ SR	—	34.3	33.0	

Obese kids in the family (%)				
None	—	29.8	55.6	**<0.001**
1-2	—	45.6	35.6	
3+	—	24.6	8.8	

Parent obesity (%)				
None obese	—	49.2	59.4	**0.002**
Obese mother	—	22.4	21.0	
Obese father	—	12.6	11.5	
Both are obese	—	15.7	8.0	

Weight-loss attempt (%)				
No attempt	—	16.5	52.4	**<0.001**
Tried but failed	—	13.8	5.7	
Tried and succeeded	—	69.7	41.9	

Data are given as mean ± standard deviation or percentage; ^*∗*^*T*-test for independent samples or chi-square tests for the proportion; SR = Saudi Riyal (1 U$ = 3.75 SR).

**Table 2:** The proportions (%) of perceived and desired body size scores selected by Saudi females with and without overweight/obesity relative to the 9 FRS scores and the normative corresponding and actual body mass index (BMI) values.

FRS	Normative corresponding BMI values^*∗*^	Perceived body size score				Desired body size score			
		Overweight/obese		Nonoverweight/nonobese		Overweight/obese		Nonoverweight/nonobese	
		%	BMI	%	BMI	%	BMI	%	BMI
1	18.3	1.6	27.4	6.6	18.3	2.4	29.9	5.5	20.9
2	19.3	5.1	28.1	30.0	19.7	34.3	28.4	38.4	21.0
3	20.9	5.9	26.8	29.5	21.1	40.9	29.5	41.3	20.9
4	23.1	23.6	27.8	27.0	22.2	20.5	30.4	12.1	20.5
5	26.2	26.8	27.8	4.9	22.2	1.2	31.1	2.2	20.4
6	29.9	22.8	30.3	1.7	22.4	0.4	29.9	0.2	21.5
7	34.3	9.4	34.0	0.3	20.5	0.3	30.7	0.2	23.1
8	38.6	4.3	36.9	0.0	—	0.0	—	0.0	—
9	45.4	0.5	44.1	0.0	—	0.0	—	0.1	23.8
Total		100		100		100		100	
**>5**		37%		4%		0.7%		0.5%	
*p* value^*∗∗*^		**<0.001**				**0.027**			

^*∗*^Corresponding BMI values were from [35]; ^*∗∗*^chi-square tests for the differences in proportions between females with overweight/obesity and those without overweight/obesity; FRS = Stunkard Figure Rating Scale.

**Table 3:** Perceived and desired body size scores, actual BMI, and some selected computed discrepancy scores among Saudi females with and without overweight/obesity.

Variable	All	Overweight/obese	Nonoverweight/nonobese	*p* value^*∗*^
Perceived body size score	3.6 ± 1.5	5.0 ± 1.5	3.0 ± 1.1	**<0.001**
Desired body size score	2.7 ± 0.89	2.9 ± 0.88	2.7 ± 0.90	**0.007**
Actual (measured) BMI	23.3 ± 5.1	29.4 ± 5.1	20.9 ± 2.4	**<0.001**
BMI corresponding to perceived body size score^*∗∗*^	22.9 ± 1.5	26.9 ± 4.9	21.3 ± 2.4	**<0.001**
BMI corresponding to desired body size score^*∗∗*^	20.7 ± 1.9	20.9 ± 1.8	20.6 ± 1.9	**0.033**
Difference between perceived and desired body size scores	−0.82 ± 1.5	−2.1 ± 1.4	−0.31 ± 1.3	**<0.01**
*Percent difference* (%)	−25.0	−42.0	−10.0	
Difference between BMI corresponding to perceived and desired body size scores	2.2 ± 4.1	−6.0 ± 4.6	−0.70 ± 2.8	**<0.01**
*Percent difference* (%)	−9.6	−22.3	−3.3	
Difference between actual BMI and BMI corresponding to perceived body size score	−0.40 ± 3.6	−2.5 ± 5.1	+0.40 ± 2.5	**<0.001**
*Percent difference* (%)	−1.7	−8.5	+1.9	
Difference between actual BMI and BMI corresponding to desired body size scores	−2.6 ± 5.3	−8.5 ± 5.2	−0.30 ± 3.1	**<0.001**
*Percent difference* (%)	−11.2	−28.9	−1.4	

^*∗*^ *T*-test for independent samples; ^*∗∗*^based on [35].

**Table 4:** Results of multiple linear regression analyses for the prediction of selected dependent variables.

Dependent variable	Predictor variables^*∗*^	Standardized coefficient (beta)	*p* value	*R* ^2^
Perceived body size scores				
Model 1	Body mass index	0.679	<0.001	0.461
Model 2	Body mass index	0.587	<0.001	0.514
	Weight-loss attempt	0.248	<0.001	

Discrepancy between perceived and desired body size scores				
Model 1	Body mass index	0.622	<0.001	0.387
Model 2	Body mass index	0.511	<0.001	0.465
	Weight-loss attempt	0.301	<0.001	

Desired body size scores				
Model 1	Body weight	0.104	0.002	0.011
Model 2	Body weight	0.136	<0.001	0.019
	Weight-loss attempt	−0.095	0.007	

^*∗*^Variables entered into the prediction equation (step-wise method): age, weight, height, BMI, father education, mother education, family income, weight-loss attempt, number of obese kids in the family, and parents' obesity.
